# Development and assessment of a website presenting evidence-based information for people with multiple sclerosis: the IN-DEEP project

**DOI:** 10.1186/s12883-016-0552-0

**Published:** 2016-03-02

**Authors:** Cinzia Colombo, Graziella Filippini, Anneliese Synnot, Sophie Hill, Roberta Guglielmino, Silvia Traversa, Paolo Confalonieri, Paola Mosconi, Irene Tramacere

**Affiliations:** Department of Public Health, Laboratory for medical research and consumer involvement, IRCCS Mario Negri Institute for Pharmacological Research, via la Masa 19, 20156 Milan, Italy; Scientific Direction, Neurological Institute C. Besta IRCCS Foundation, via G. Celoria 11, 20133 Milan, Italy; Cochrane Multiple Sclerosis and Rare Diseases of the Central Nervous System Review Group, Neurological Institute C. Besta IRCCS Foundation, via G. Celoria 11, 20133 Milan, Italy; Centre for Health Communication and Participation, School of Psychology and Public Health, College of Science, Health and Engineering, La Trobe University, Plenty Road & Kingsbury Drive, Melbourne, Victoria 3086 Australia; National Trauma Research Institute, Monash University/The Alfred, Level 4, 89 Commercial Road Melbourne, Melbourne, 3004 Victoria Australia; Scientific Research Area, Italian Multiple Sclerosis Foundation (FISM), Via Operai 40, 16149 Genoa, Italy; Department of Neuroimmunology, Neurological Institute C. Besta IRCCS Foundation, Via G. Celoria 11, 20133 Milan, Italy; Unit of Neuroepidemiology, Cochrane Multiple Sclerosis and Rare Diseases of the Central Nervous System Review Group, Neurological Institute C. Besta IRCCS Foundation, Via G. Celoria 11, 20133 Milan, Italy

**Keywords:** Multiple sclerosis, Consumer health information, Internet, Information dissemination, Patient information needs

## Abstract

**Background:**

People with multiple sclerosis (MS) are increasingly using the Internet in the daily management of their condition. They search for high-quality information in plain language, from independent sources, based on reliable and up-to-date evidence. The Integrating and Deriving Evidence, Experiences and Preferences (IN-DEEP) project in Italy and Australia aimed to provide people with MS and family members with an online source of evidence-based information, starting from their information needs. This paper reports on the Italian project’s website.

**Methods:**

Contents, layout and wording were developed with people with MS and pilot-tested. The website was evaluated using an online 29-item questionnaire for ease of language, contents, navigation, and usefulness of information aimed at people with MS, family members and the general population.

**Results:**

The website (http://indeep.istituto-besta.it/) is structured in multiple levels of information. The first topic was interferons-β for people with relapsing-remitting MS. In all, 433 people responded to the survey (276 people with MS, 68 family members and 89 others). The mean age was 45 years, almost 90 % had a high school diploma, about 80 % had relapsing-remitting MS, and the median disease duration was seven years. About 90 % judged the website clear, understandable, useful, and easy to navigate. Ninety percent of people with MS and family members would recommend it to others. Sixty-two percent reported they felt confident in making decisions on interferons-β after reading the website.

**Conclusions:**

The model was judged clear and useful. It could be adapted to other topics and diseases. Clinicians may find it useful in their relationship with patients.

**Electronic supplementary material:**

The online version of this article (doi:10.1186/s12883-016-0552-0) contains supplementary material, which is available to authorized users.

## Background

*"I think there are too many confusing things on the Internet. I don’t know how much to trust of the information that I find. I need true, easy-to understand health information that is useful to meet my needs".* (A person with multiple sclerosis participating in the IN-DEEP focus group)

People with multiple sclerosis (MS) are mainly young adults, who are increasingly using the Internet for MS-related information. While neurologists or general practitioners remain the principal or at least the most trusted source of information for most people, the Internet is often used to supplement their advice. People with MS use the Internet to search for health information, stay abreast of research, check claims to treatment benefits and adverse effects reported in the media and social networks [1–3].

A search on Google® (in English) in June, 5th 2015 using the search term “multiple sclerosis” retrieved 23.4 million links. From this huge amount of information, people need to know how to assess the relevance of the information and how it relates to them personally [4–6]. In a recent study exploring the online health information needs and barriers for people with chronic health conditions, participants complained of differences in the information from different online sources as a recurrent difficulty, and stated their desire for health professionals to play a role in guiding them to find relevant and reliable online health information [7]. For people with MS to self-manage their condition, information has to be of high quality, in plain language, from independent sources, and based on reliable and up-to-date evidence [1, 8–11].

**Fig. 1:**
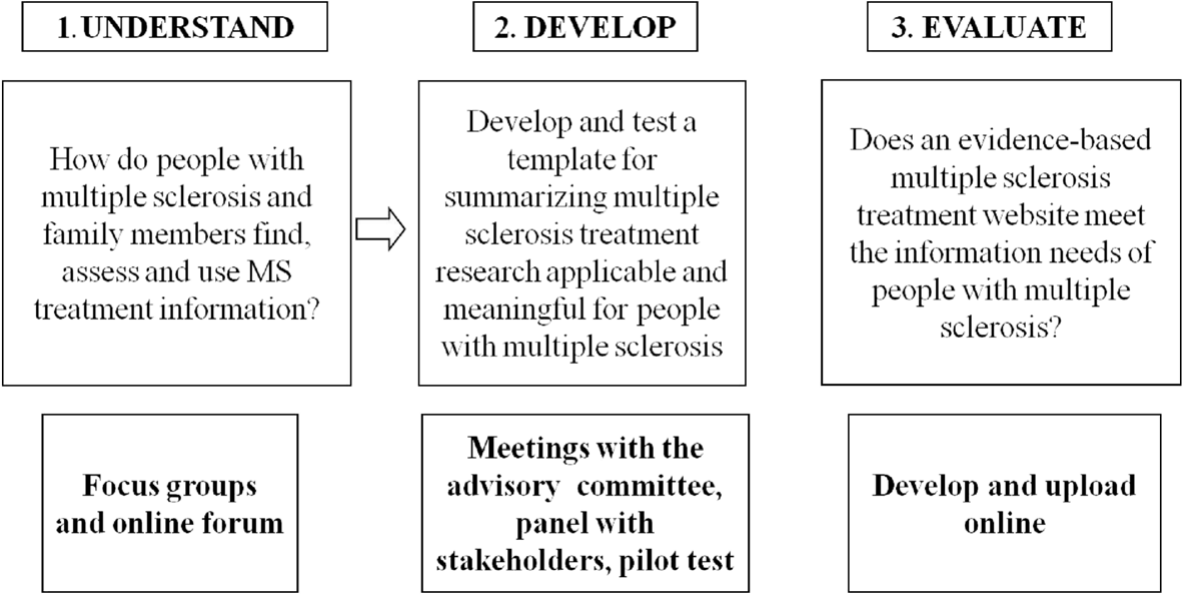
The steps of the IN-DEEP project

The Integrating and Deriving Evidence, Experiences and Preferences (IN-DEEP) project is an Italian-Australian collaboration developing two parallel projects to explore how people with MS integrate health information they find on the Internet with their needs, experiences, preferences and values, and how these factors could be incorporated into an online source of evidence-based information that was accessible and meaningful to them and to family members of people with MS [12]. It is an informational intervention aimed to provide qualified information through the web, to support the decision-making of people with MS with clinicians. A four-stage process was used: first, health information needs were assessed through focus groups with people with MS and family members led by a psychologist [13, 14]; second, a template was developed presenting evidence-based health information; third, the template was implemented on the web and fourth, an online survey was conducted to assess this web-based resource (Fig. 1).

Most of the people with MS and family members who were involved in the focus groups during the first phase of the Italian IN-DEEP project [13] reported difficulty navigating through the mass of Internet data, in understanding and evaluating quality and reliability of web information. They said that web information often does not cover their information needs or is inappropriate. There was a general perception that good quality independent web information is lacking. Even so, participants concluded that the Internet was useful for people with MS. Their information needs covered comprehensive communication of diagnosis, prognosis, benefit and adverse events of treatments, and new drugs, gradually changing over the course of the disease.

As reported in the literature, informational interventions aimed at improving health literacy, in conjunction with medical advice, can improve people’s knowledge, symptom management and treatment adherence, reduce anxiety, and increase a sense of empowerment. In particular, websites have beneficial effects on self-efficacy and health behavior [15].

This paper describes the development of the Italian IN-DEEP website and its assessment through an online survey.

## Methods

The project was a collaboration between the Cochrane Multiple Sclerosis Group - Fondazione IRCCS Istituto Neurologico “Carlo Besta”, Milan, IRCCS- Istituto di Ricerche Farmacologiche “Mario Negri” Milan, the Centre for Health Communication and Participation, La Trobe University, Melbourne, and Italian and Australian MS societies. It was approved by the Faculty of Health Sciences Human Research Ethics Committee of La Trobe University, Australia, and the Ethics Committee of the Fondazione IRCCS Istituto Neurologico Carlo Besta, Milan, Italy.

The project’s advisory committee included neurologists, people with MS, experts in health literacy and communication and representatives of the Italian MS society. All the phases of the project were discussed and shared with the advisory committee.

### The website

The website was developed through four main phases.

1. Development of a template
  The purpose, structure and type of information covered were decided on the basis of the focus group findings [13] and the literature available [16–18] and then discussed with the advisory committee. The first topic was treatment with interferons-β (IFNs) for people with relapsing-remitting MS (RR-MS). It was selected as a first example to test the information model considering that IFNs are used widely in clinical practice and relevant scientific literature is available.
  People with MS also asked for personal stories, considered useful to convey and reinforce the messages and translate them into daily life [13]. A convenience sample of personal stories of people with MS was collected by the neurologists involved in the advisory committee. The stories were based on three questions: 1. What was the effect of being diagnosed with multiple sclerosis in your personal experience? 2. Which questions did you ask yourself to decide about taking interferon? Which information did you need and look for? 3. How has been your daily life taking interferon? Did interferon therapy change your life?
  The stories-literally reported-accompany the main body of the website as an insight of patients voice on their personal experience using interferon, and the decision process to take it in terms of information needs.
2. Review panels with people with MS
  The structure of the template, content, layout and wording were discussed with people with MS and communication experts. Cochrane reviews were used as the main evidence-based source for IFNs benefit and short-term adverse events [19]. Information on medium and long-term adverse events, not available from randomised trials included in the Cochrane reviews, was extracted from other sources, after checking the quality of primary studies. Layout and format for presenting benefits and harms of IFNs were first developed on the basis of the literature [20–22] then discussed in face-to-face interviews with people with MS (n.9).
3. Website development and pilot testing
  The final draft was adapted for the web and put online for a short pre-test phase, whereby it was pilot tested with a convenience sample of four clinicians, three people with MS, four experts in communication and web design and three lay people.
4. Website revisions and uploading
  The final version was hosted on the website of the Fondazione IRCCS Istituto Neurologico “Carlo Besta”, Milan, Italy [23] and launched at the end of October 2012.

### The survey

The website was evaluated using an online survey promoted through a press release, articles on the websites and newsletters of the partners of the project, invitations by e-mail, lay press articles, and presentations at meetings reaching the general public, representatives of patient and citizen associations, people with MS, clinicians, and researchers.

The 29-item questionnaire was informed by relevant literature [17, 18]; before being reviewed by the advisory committee and refined again after pilot testing (see Additional file 1).

General questions were directed to all respondents, asking if the language and wording were clear, the contents easy to understand, the information useful, and the website easy to navigate. Specific questions directed to people with MS and family members only related to risks and benefits of IFNs treatment in RR-MS, satisfaction with the online resource, whether it met respondents’ needs, and its usefulness in making decisions about therapy. The survey was open for 4 months (November 2012 to February 2013) and participation was anonymous. Information about the project and the survey- i.e., length of time of the survey, which data were stored and where, the anonymity of responses, who the investigators were, the purpose of the study– was reported in the IN-DEEP section “About us” and on the page hosting the online survey. Filling the questionnaire was considered an implied consent to participate to the survey.

#### Statistical analyses

Demographic and clinical characteristics of the sample were analysed as percentages for categorical data, means with standard deviations and medians with the corresponding range for continuous variables, and by group (people with MS, family members and others). The distribution of the sample’s answers regarding language and wording, comprehensibility of contents, usefulness of information – in general and specific for risks and benefits of IFNs in RR-MS-and ease of web navigation is reported.

## Results

### The website

**Fig. 2:**
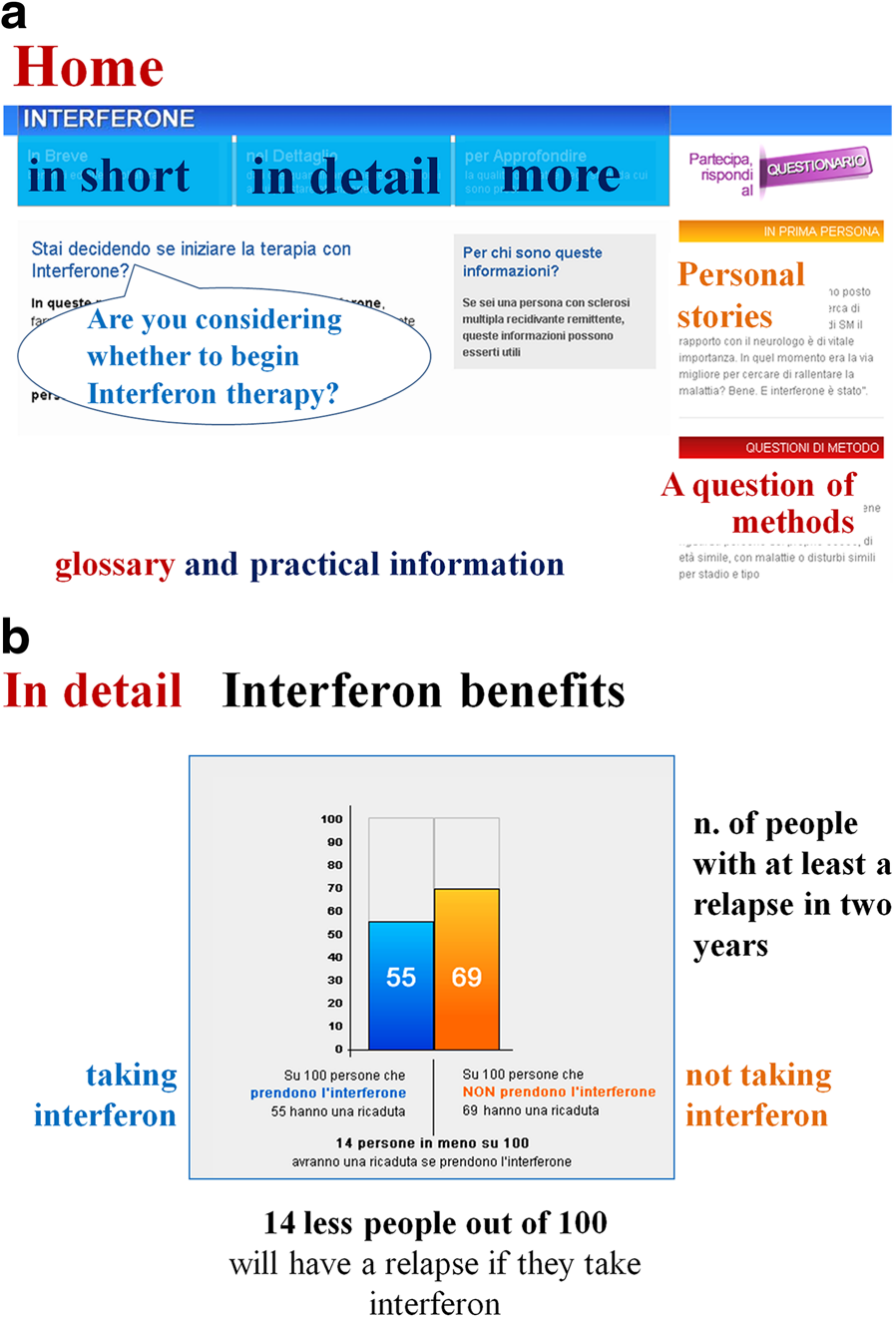
**a** IN-DEEP home page. **b** Graphic presentation of a selection of benefits of interferons

Considering that the information needs of people with MS gradually change over the course of the disease [13, 14] the website reflected preferences for information layered in three levels- “in short” “in detail”, “to know more” (Fig. 2a). Benefits of IFNs were reported in the three levels of detail, with a few phrases in the section “in short”, numerical data and graphs in the section “in detail”, and information about the sources in “to know more” section. Bar graphs were used to illustrate numerical data of the IFNs benefits (Fig. 2b), as they were considered clearer than other layouts (e.g., icons) by the people with MS who were interviewed. Harms were reported in a table divided by frequency, without detailed numerical data, and by type of IFN, i.e., Avonex, Rebif and Betaferon.

The need for qualified information expressed by the focus groups was addressed by selecting evidence-based sources of information where available, and citing the sources linked to a methodological section explaining the strength of evidence of different types of studies (e.g., randomized controlled trials, systematic reviews). Two sections called “what we know for sure” and “what we do not know for sure yet” distinguished information from strong evidence-based sources (short-term benefits) from the areas of uncertainty still present in the literature (mean long-term effects and when to give up IFNs). Information on the long-term adverse effects of IFNs, a topic raised by people with MS in the focus groups and the working group, was extracted from sources such as European Medicines Agency (EMA) reports [24–26] and the Micromedex database [27], and checked against primary studies.

The difficulties in assessing the quality of web-based health information arising from the focus groups suggested the need for educational tools such as a glossary and tools to critically assess health information websites and health information in general (“Misura-siti”, “Misura-informazione”) [28].

A section was dedicated to the personal stories of people with MS related to the topic covered (e.g., “how I decided to start treatment with IFN” or “my experience with IFN treatment”). To address people with MS’ need to translate online information to their own condition, a section called “Is this information useful for me?” described the participants in clinical trials with IFNs and explained how their characteristics can be applied generally. A list of questions to ask to their neurologist, and practical information on IFNs treatment (e.g., how to injections, to bring it medication on flights) was also provided.

### The survey

In total, 555 participants started the survey, and 433 (70 %) completed the survey in full.

**Fig. 3:**
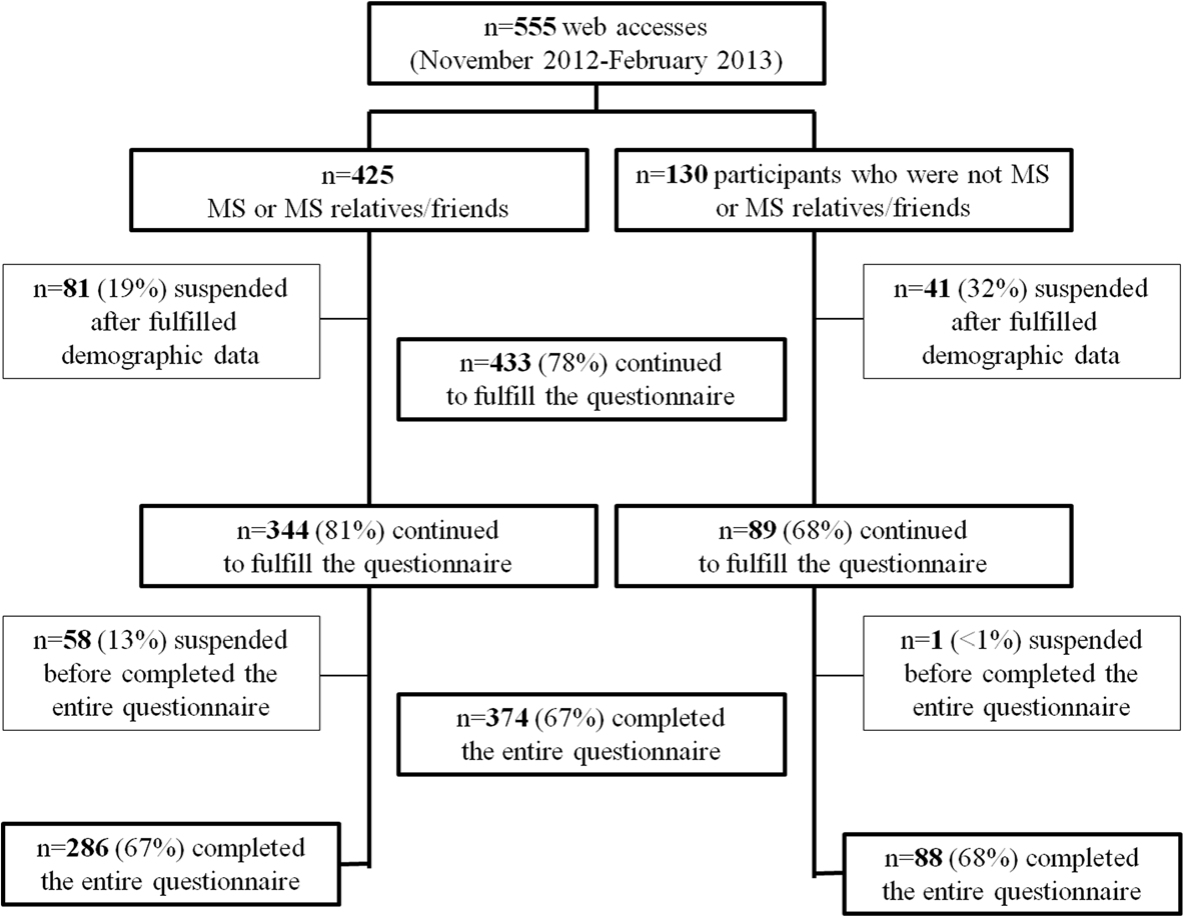
Flow-chart

Of 555 web accesses, 425 were from people with MS or family members and 130 from the general population. Survey profile is reported in Fig. 3. Clinical and demographic characteristics of participants who only provided demographic data were similar to those who completed a part or all of the questionnaire (data not shown). In all, 433 questionnaires were analysed (Table 1).

**Table 1:** Clinical and demographic characteristics of the sample

	People with MS	Family members of people with MS	Other	Overall
	(276)	(68)	(89)	(433)
	*n*. (%)	*n*. (%)	*n*. (%)	*n*. (%)
Diagnosis				
Primary progressive	12 (5)	3 (7)	-	15 (5)
Secondary progressive	32 (13)	13 (31)	-	45 (16)
Relapsing remitting	203 (82)	26 (62)	-	229 (79)
Missing data	29	26	-	55
Duration of disease from onset of symptoms (years)				
Mean ± SD	9 ± 8	9 ± 9	-	9 ± 8
Median (range)	7 (0–32)	6 (0–32)	-	6 (0–32)
Missing data	0	4	-	4
Sex				
Female	192 (70)	48 (71)	63 (71)	303 (70)
Male	84 (30)	20 (29)	26 (29)	130 (30)
Age (years)				
Mean ± SD	43 ± 11	45 ± 12	49 ± 13	45 ± 12
Median (range)	43 (2072)	45 (21–72)	50 (23–76)	45 (20–76)
Area of residence				
Northern Italy	138 (50)	42 (62)	58 (66)	238 (55)
Central Italy	72 (26)	22 (16)	15 (17)	98 (23)
South and islands	65 (24)	15 (22)	15 (17)	95 (22)
Missing data	1	0	1	2
Size of places of residence				
< 5000 inhabitants	49 (18)	16 (24)	10 (11)	75 (17)
5000–50,000 inhabitants	116 (42)	22 (32)	23 (26)	161 (37)
50,000–100,000 inhabitants	30 (11)	14 (21)	7 (8)	51 (12)
100,000–500,000 inhabitants	42 (15)	5 (7)	13 (15)	60 (14)
> 500,000 inhabitants	38 (14)	11 (16)	35 (40)	84 (19)
Missing data	1	0	1	2
Education				
Primary school diploma	2 (1)	2 (3)	1 (1)	5 (1)
Middle school diploma	30 (11)	10 (15)	14 (16)	54 (12)
High school diploma or higher	244 (88)	56 (82)	74 (83)	374 (87)
Employment				
Employed	185 (67)	50 (74)	48 (54)	283 (65)
Retired	33 (12)	9 (13)	26 (29)	68 (16)
Student	10 (4)	1 (1)	2 (2)	13 (3)
Homemaker	22 (8)	1 (1)	8 (9)	31 (7)
Unemployed looking for work	26 (9)	7 (10)	5 (6)	38 (9)

*Abbreviations*: *MS* multiple sclerosis

Most were RR-MS, the duration of disease varied from 0 to 32 years (median 7). More than two third were females, with a mean age of 45 years. More than a half lived in small towns in northern Italy. A high proportion had a high school diploma or over and two-thirds were employed. No significant difference emerged in clinical characteristics (respondent people with MS compared to people with MS relatives of respondent family members) or other main characteristics (people with MS, family members, general population).

No significant differences in the distribution of answers were observed by group for the general questions aimed at the overall respondents (people with MS, family members and general population) nor for specific questions aimed at people with MS and family members (data not shown).

Table 2 illustrates the perception of the website in terms of plain language, comprehensibility of contents, usefulness of information, and ease of navigation. Most of the respondents gave positive judgments. Two percent of respondents or less gave negative answers.

**Table 2:** Survey findings. Selection of items

	No/Not really	Somewhat	Really/extremely
	*n*. (%)	*n*. (%)	*n*. (%)
Overall sample n.433			
Is the website easy to navigate?	8 (2)	23 (5)	387 (93)
Is the information easy to understand?	3 (1)	54 (12)	376 (87)
Is the information useful?	9 (2)	59 (14)	365 (84)
Persons with MS and family members n. 344^a^			
Are the benefits of interferon clear?	18 (6)	57 (19)	229 (75)
Are the harms of interferon clear?	13 (4)	36 (12)	255 (84)
Are graphic presentations of IFN benefits easy to understand?	8 (3 %)	54 (18 %)	242 (79 %)
Are tables of IFN harms easy to understand?	8 (3 %)	37 (12 %)	259 (85 %)
Is the information on benefits and harms useful?	11 (4)	59 (21)	216 (75)
Are you more confident in making decisions about interferon therapy?	26 (9)	82 (29)	178 (62)

*Abbreviations*: *IFN* interferon, *MS* multiple sclerosis

^a^The sum does not add up to the total because of some missing values. The percentages are calculated on the total responders to the single question

The three-level data reporting (“in short” “in detail” “to know more”-Fig. 2a) was judged to be extremely useful in order to understand information by 80 % of respondents. In addition, 90 % said they would recommend the IN-DEEP website to people with MS (8 % responded “don’t know” and 2 % “no”).

Most people with MS and family members considered the information on benefits and harms of IFNs really/extremely useful and their graphic presentations very clear (75 %) and easy to understand, while 6 % considered the benefits, and 4 % the harms not clear. Twenty-nine percent reported they were somewhat and 62 % really/extremely confident in making decisions about IFNs therapy (Table 2).

Information in the section “to know more”, dedicated to methodological aspects of studies, and in the glossary was judged very useful and clear by people with MS and family members (more than 85 % of really/extremely and less than 3 % of no/not really).

In addition, 79 % of people with MS and family members answered that the IN-DEEP online resource met their information needs, 17 % moderately and only 5 % no/not really. Free comments frequently mentioned that people with MS and family members wanted to find other information on the disease (causes, risk factors, diagnostic tests, prognosis), on other MS forms and on other in treatments used (data not shown).

## Discussion

The IN-DEEP website is shaped on the needs and feedback of people with MS and family members and is structured in multiple levels of depth so the reader can decide how much and what kind of information to read. It represents an information model that fosters an active role for people with MS and family members in searching for information to make healthcare decisions. The principles of this model are summarized as follows: developing the structure and contents starting from the needs of people with MS; providing multiple-level evidence-based information with words, numbers and pictures; citing the sources and describing their quality; presenting methodological information and tools to critically appraise health information; presenting tools to apply the information to the personal condition of the reader, including personal stories. Most of these principles reflect the criteria for an evidence-based information for patients reported in the literature [16].

As reported in a recent Cochrane review [29], informational interventions for people with MS are various, cover different topics with various approaches and outcomes. They seem to increase the knowledge of people with MS about topics related to the disease, without conclusive results on decision making and quality of life. We assessed the IN-DEEP website in terms of comprehensibility and usefulness, asking people with MS if they would have used it in making decisions and if it responded to their needs. Most respondents judged it clear and useful. The overall sample reported positive judgments, with almost always at least 80 % of high and very high and no more than 5 % of low and very low opinions. The majority reported they would recommend the IN-DEEP website to others, but a few found it less useful to make decisions. One reason could be that some topics were not covered. People with MS asked for plain information about drug comparisons, new treatments and diagnostic test results, not yet covered by the IN-DEEP website. The other reason could be that respondents rely on the neurologist to make decisions related to therapies. Free-text comments included, “the decision about interferon treatment is made by the neurologist. It is a proposal which patients can complain about: medical expertise is different from information”; and “for this kind of delicate decision I prefer to trust the judgment of the neurologist, within a good doctor-patient relationship based on trust”. The relationship with a trusted neurologist is the first reference point for decisions. This is in line with the findings of an Italian study about the attitudes of people with MS towards medical decision making in clinical setting, showing that the majority of people involved preferred a collaborative role (decision with neurologist), followed by a passive role (decision by neurologist), and finally by an active one (have the last say) [30].

We found in earlier focus groups that patients refer to the web without telling the neurologist when their information needs remain unanswered, either because of the lack of time during a visit or the neurologist’s difficulty in grasping the patients’ requests. A recent study [31] showed that Italian MS physicians need more training on shared decision making skills, to understand patient preferences for reception of information and involvement in health decisions. We suggest that neurologists may find the IN-DEEP website a useful tool to foster a dialogue with patients about the therapy.

We drew together evidence-based MS information with the needs of people with MS: the challenge was to match people with MS’ information needs with the best sources available from research. The mismatch between patients needs and research is well documented [32] and is an issue also in the dissemination of the information. We tried to tackle by it including information from various sources clearly explaining their limits and the related uncertainty. As reported in literature [33], people with MS are not scared by complex evidence-based information showing the uncertainties of data.

Answering the information needs of patients in a situation of uncertainty is a difficult task even for clinicians during the visit. This is why clinicians may find a web-based resource that was developed starting from people with MS’ needs and with their cooperation helpful [33, 34]. Time constraints often limit the amount and kind of information that clinicians provide to patients. A website such as IN-DEEP could be suggested by clinicians as a source of high-quality research about treatment for MS, that is potentially useful also to increase patients’ critical appraisal skills. The application of the IN-DEEP website in this context should be implemented and assessed widening the topics covered.

### Strengths and limitations

Considering the lack of data about long term adverse effects of interferons from clinical trials included in the Cochrane review, we collected data from the EMA reports and the Micromedex database in order to answer people with MS’ information needs, indicating the methodological limits of these sources.

As for the survey, the sample was representative of people with MS and the population distribution of Italians, as reported in the literature [35].

We lost 20 % of the sample who only provided demographic data, but we found no differences in the demographic and clinical characteristics of this sample compared to the other responders, suggesting the results are robust.

The level of education of respondents to the survey was high, compared to that reported by the Italian National Institute of Statistics (ISTAT) [36]. According to the ISTAT report, in Italy 58 % of people, from 25 to 64 years of age, have a high school diploma or over, with larger proportions in central (63.7 %) and northern (61.3 %) Italy. In our survey, 87 % of respondents had corresponding similar level of education, with no significant difference by region [data not shown]. A high level of education of respondents was expected, since this was an online survey [37, 38] and Internet use is driven partly by educational level [39] but the generalisability of the results, especially regarding the readability and understandability of the information, need to be specifically assessed with people with lower education levels.

Since the aim of the survey was to test a web-based model, results refer to persons who used the internet. This is in line with the increasing use of the web by people with MS to search for information related to the disease.

Information on interferons presented in the website is one piece of the information jigsaw. The personal stories were added as an example of another piece of information, i.e., patients’ preferences and experiences. In the framework of the IN-DEEP website, we included also the methodological section and the section dealing with the critical appraisal of health information, both underlying the different levels of certainty deriving from different kind of sources (case reports, controlled trials, randomized controlled trials, systematic reviews…).

Finally, interferons for relapsing-remitting multiple sclerosis are the first example used to test the information model. Other topics are under development.

## Conclusions

The IN-DEEP website meets the need of people with MS for good-quality information, and we shall use this model as a basis for developing further topics of relevance to them.

The IN-DEEP web-based model could be adapted to other diseases and conditions, in the framework of collaboration among clinicians, researchers, experts in communication and patient associations.

### Availability of data and materials

The data related to the survey are stored at the Neurological Institute C. Besta IRCCS Foundation and are available under request.

## Additional file

Additional file 1: Appendix 1. Questionnaire. (PDF 23 kb)

## Abbreviations

MS: multiple sclerosis
IN-DEEP: integrating and deriving evidence, experiences and preferences (project)
IFNs: interferons
RR-MS: relapsing-remitting multiple sclerosis
EMA: European Medicines Agency
ISTAT: Italian National Institute of Statistics
