# Supplementary material for: Development and assessment of a website presenting evidence-based information for people with multiple sclerosis: the IN-DEEP project
Source: BMC Neurol. 2016 Mar 2;16:30. doi: 10.1186/s12883-016-0552-0 (PMC4776365; doi:10.1186/s12883-016-0552-0)
Supplement: Additional file 1: — Appendix 1. Questionnaire. (PDF 23 kb) [file 12883_2016_552_MOESM1_ESM.pdf]

Appendix 1. Questionnaire

1. How did you know the IN-DEEP website?

- o from another website
- o an invitation by e-mail
- o from Facebook
- o suggestion by relative or friend
- o other, please specify…..

2. Female ☐ Male ☐

3. Age

4. What is your home postcode? ________________________________

5. Tick at least one: You are....

- o A person with MS

    When have you been diagnosed with MS? ____________(year)

    Which type of MS do you have?

    Relapsing remitting MS

    Secondary progressive MS

    Primary progressive MS

    Progressive relapsing MS

    Don't know

- o A family member of a person with MS

    When has your family member been diagnosed with MS? ____________(year)

    Which type of MS does your family member have?

    Relapsing remitting MS

    Secondary progressive MS

    Primary progressive MS

    Progressive relapsing MS

    Don't know

- None of the options above

6. What is the highest level of education you have attended? (Tick one only)

- Primary school diploma
- Middle school diploma
- Senior high school
- University
- No diploma

7. What is your occupation? (Tick one only)

- Employed
- Unemployed looking for work student
- Homemaker
- Retired

8. How often would you usually use a computer for Internet activities (both at home and at work)?

- Less than once a month
- Some times a month
- At least once a week
- Most days of the week
- Every day

In general, the information reported on the IN-DEEP website is

	Not at all	A little bit	Somewhat	Really/extremely
9. Easy to read				
10. Easy to understand				
11.Useful				
12. Reliable				

Please, tell us why…

	Not at all	A little bit	Somewhat	Really/extremely
13. Is the information in the sections “A question of method” and “Glossary” useful?				
14. The three-level reporting (“in brief”, “in details”, “more”) makes the information easy to be read?				
15. Do you think the three levels should be maintained?				
16. Is the IN-DEEP website easy to navigate?				

17. Would you suggest IN-DEEP to other people with MS or families?

Yes No Don’t know

Please give us your comments

If you are a person with MS or a family member, please go on and fill the 10 questions below.

Section “In detail“

	Not at all	A little bit	Somewhat	Really/extremely
19. Is the information on the benefits of IFN clear?				
20. Is the graphical presentation of the benefits of IFN clear?				
21. Is the information on harms of IFN clear?				
22. Is the table reporting the harms of IFN clear?				

23. Is the information in this section useful?				

Section “More”

	Not at all	A little bit	Somewhat	Really/extremely
24. Is the information in this section clear?				
25. Is the information in this section useful?				

In general

	Not at all	A little bit	Somewhat	Really/extremely
26. Does the information in the IN-DEEP website responded to your information needs?				
27. Is the information on benefits and harms of IFN useful?				
28. Do you feel more confident in making decisions about IFN?				
29. Would you use the information in the IN-DEEP website to make decisions about IFN?				

Please give us your comments
